# Differentiation of periapical granulomas and cysts by using dental MRI: a pilot study

**DOI:** 10.1038/s41368-018-0017-y

**Published:** 2018-05-17

**Authors:** Alexander Juerchott, Thorsten Pfefferle, Christa Flechtenmacher, Johannes Mente, Martin Bendszus, Sabine Heiland, Tim Hilgenfeld

**Affiliations:** 1 Department of Neuroradiology, Heidelberg University Hospital, Im Neuenheimer Feld 400, Heidelberg, Germany; 2 Division of Endodontics and Dental Traumatology, Department of Conservative Dentistry, Heidelberg University Hospital, Im Neuenheimer Feld 400, Heidelberg, Germany; 3 Institute of Pathology, Heidelberg University Hospital, Im Neuenheimer Feld 224, Heidelberg, Germany; 4 Division of Experimental Radiology, Department of Neuroradiology, Heidelberg University Hospital, Im Neuenheimer Feld 400, Heidelberg, Germany

## Abstract

The purpose of this pilot study was to evaluate whether periapical granulomas can be differentiated from periapical cysts in vivo by using dental magnetic resonance imaging (MRI). Prior to apicoectomy, 11 patients with radiographically confirmed periapical lesions underwent dental MRI, including fat-saturated T2-weighted (T2wFS) images, non-contrast-enhanced T1-weighted images with and without fat saturation (T1w/T1wFS), and contrast-enhanced fat-saturated T1-weighted (T1wFS+C) images. Two independent observers performed structured image analysis of MRI datasets twice. A total of 15 diagnostic MRI criteria were evaluated, and histopathological results (6 granulomas and 5 cysts) were compared with MRI characteristics. Statistical analysis was performed using intraclass correlation coefficient (ICC), Cohen’s kappa (κ), Mann–Whitney *U*-test and Fisher’s exact test. Lesion identification and consecutive structured image analysis was possible on T2wFS and T1wFS+C MRI images. A high reproducibility was shown for MRI measurements of the maximum lesion diameter (intraobserver ICC = 0.996/0.998; interobserver ICC = 0.997), for the “peripheral rim” thickness (intraobserver ICC = 0.988/0.984; interobserver ICC = 0.970), and for all non-quantitative MRI criteria (intraobserver-κ = 0.990/0.995; interobserver-κ = 0.988). In accordance with histopathological results, six MRI criteria allowed for a clear differentiation between cysts and granulomas: (1) outer margin of lesion, (2) texture of “peripheral rim” in T1wFS+C, (3) texture of “lesion center” in T2wFS, (4) surrounding tissue involvement in T2wFS, (5) surrounding tissue involvement in T1wFS+C and (6) maximum “peripheral rim” thickness (all: *P* < 0.05). In conclusion, this pilot study indicates that radiation-free dental MRI enables a reliable differentiation between periapical cysts and granulomas in vivo. Thus, MRI may substantially improve treatment strategies and help to avoid unnecessary surgery in apical periodontitis.

## Dental imaging: Diagnosing tooth root lesions using magnetic resonance imaging

Magnetic resonance imaging (MRI) can differentiate between periapical (apex of a tooth root) cysts and granulomas (inflammations usually caused by infections). MRI is a radiation-free, non-invasive imaging technique, and a team headed by Tim Hilgenfeld at Heidelberg University Hospital in Germany conducted a pilot study to determine whether dental MRI could be used to distinguish periapical cysts from granulomas in 11 patients with confirmed periapical lesions. The authors found that dental MRI allowed for the identification of six characteristics, each of which had the capacity to clearly differentiate between periapical cysts and granulomas. The team’s findings need to be confirmed in future studies with larger numbers of patients. But the results could have a substantial clinical impact in improving diagnoses and avoiding unnecessary surgery in patients with periapical lesions.

## Introduction

Apical periodontitis is a chronic inflammatory disorder of periradicular tissues, usually due to bacterial infection of the root canal system.^1^

The pathogenesis of apical periodontitis and the cause of endodontic failure have been extensively reviewed by Nair et al.^2^: Microorganisms (mainly obligate anaerobes, fungi) play an essential role in this process. Their pathogenicity is influenced by the endodontic flora (microbial interaction, interference with the host immune system through microbial modulins, endotoxins and enzymes), cellular elements of the host response (polymorphonuclear neutrophils, lymphocytes, macrophages, osteoclasts and epithelial cells) and molecular mediators (cytokines, interferon, colony-stimulating factor, growth factors, eicosanoids, effector molecules and antibodies).^2^ The lack of blood circulation in the root canal system of necrotic teeth protects the microbes present within the necrotic pulpal tissue from the host’s defenses and antibiotic therapy, thereby prolonging their presence.^1^ The result is a dynamic interplay between host and microbes. The latter may spread into the periapical tissues causing periapical periodontitis. In acute cases, symptoms such as pain, tooth elevation and tenderness to pressure occur. Several outcomes are possible: spontaneous healing, exacerbation and spreading of the infection to the bone or the exterior (alveolar abscess or fistulation/sinus tract formation) and chronification.^1,3^

Chronic periapical periodontitis develops after a shift in the host immune response. The prolonged presence of microbes results in asymptomatic lesions and bone resorption.^3^ This is radiographically visible as periapical lesions although histological backgrounds can differ (granuloma or cyst).^1^

Nair et al.^4^ showed that up to 85% of all periapical lesions are granulomas. “Periapical granulomas” contain granulomatous tissues, cell infiltrates and a fibrous capsule, and are a diagnostic marker for the diagnosis of chronic apical periodontitis.^4^ Radicular cysts are considerably less frequent^4^ and occur in two distinct histological categories: (I) apical true cysts and (II) apical pocket cysts.^5^ Radicular true cysts are entirely enclosed by epithelium. They are believed to originate from dormant epithelium, which has been stimulated to proliferate. Periapical pocket cysts are lined by epithelium but are open to the root canal, effectively sealing off a pocket-like micro-abscess from the periapical surroundings. Continued enlargement of these cysts results in slow but progressive destruction of the surrounding bone and matrices.^4,5^

It is widely believed that granulomas and pocket cysts may heal after non-surgical root canal therapy,^6^ whereas true cysts are self-sustaining and therefore less likely to be resolved by non-surgical treatment. The success rates for initial orthograde treatment of teeth with chronic apical periodontitis range from 76% to 88%^7^ and for non-surgical retreatment from 71% up to 83% in the long term (4–6 years after orthograde retreatment).^8^ Where treatment providers are endodontic specialists using a dental operating microscope and performing their treatments with the orthograde placement of mineral trioxide aggregate (MTA) apical plugs, success rates of > 90% can be achieved.^9^

In cases with periapical lesions measuring ≥ 5 mm in size, success rates appear to be lower for primary treatments (66.9%) and drop to 53.3% for orthograde retreatments.^10^ Success rates of 80.6% have been reported for teeth with periapical lesions > 5 mm for treatments performed by an endodontic specialist.^11^ Furthermore, larger lesions are more likely to be radicular cysts^12^ associated with lower success rates for (non-surgical) orthograde treatment or retreatment.^10^ Success rates of 59% up to 94% can be achieved by apical surgery for these teeth. Treatment outcome highly depends on the technique used (traditional or modern). Modern techniques incorporating microsurgical instruments and high-power magnification are much more successful.^13^ However, the risks associated with apical surgery, such as damage to nerves, soft tissue, bone or neighboring teeth, as well as hemorrhage, have to be considered in the context of the patient’s general health status.^14^

A non-invasive diagnostic method for a reliable in vivo lesion characterization (apical granuloma or radicular cyst) would be of great clinical value in order to minimize treatment-associated risks, costs and patient discomfort. This would be particularly valuable in cases with periapical lesions ≥ 5 mm, where the optimal treatments for granulomas and cysts differ: non-invasive orthograde (re)treatment for granulomas and invasive apical surgery for cysts. To date, however, such a tool is not available, since previous diagnostic approaches including periapical radiographs,^15,16^ roentgenographic contrast medium,^17^ Papanicolaou smears,^18^ electrophoresis^19^ and cone-beam computed tomography (CBCT)^20,21^ have proven to be unreliable. Thus, surgical biopsy and subsequent histopathological evaluation have remained the gold standard for confirming the diagnosis of periapical lesions.^22^

Magnetic resonance imaging (MRI) is a radiation-free imaging modality characterized by excellent soft tissue contrast. Technical advances associated with the use of higher field strength,^23^ dedicated coil systems^24,25^ and optimized sequence techniques^26–28^ have led to an improved image quality followed by an increased interest in dental MRI. Feasibility studies in the fields of implantology,^29^ orthodontics,^30^ periodontology^31^ and endodontics,^32,33^ for instance have shown promising results. As a result of recent technical developments, MRI has become a promising method for characterizing periapical lesions.^34,35^

Nevertheless, it is still unclear, whether periapical cysts can be distinguished from periapical granulomas by using MRI. For this reason, we aimed to investigate whether dental MRI allows for a reliable differentiation between periapical cysts and granulomas. For the first time, we compared structured analysis of non-contrast-enhanced (T1- and T2-weighted), as well as contrast-enhanced (T1-weighted) MRI images with histopathological results.

## Results

Histopathological analysis of the 11 periapical lesions included in the study revealed six granulomas and five cysts. One cyst was a keratocyst, all other cysts were radicular cysts.

All periapical lesions included were detectable on MRI and there were no relevant alterations of image quality due to motion or metal artifacts.

**Fig. 1:**
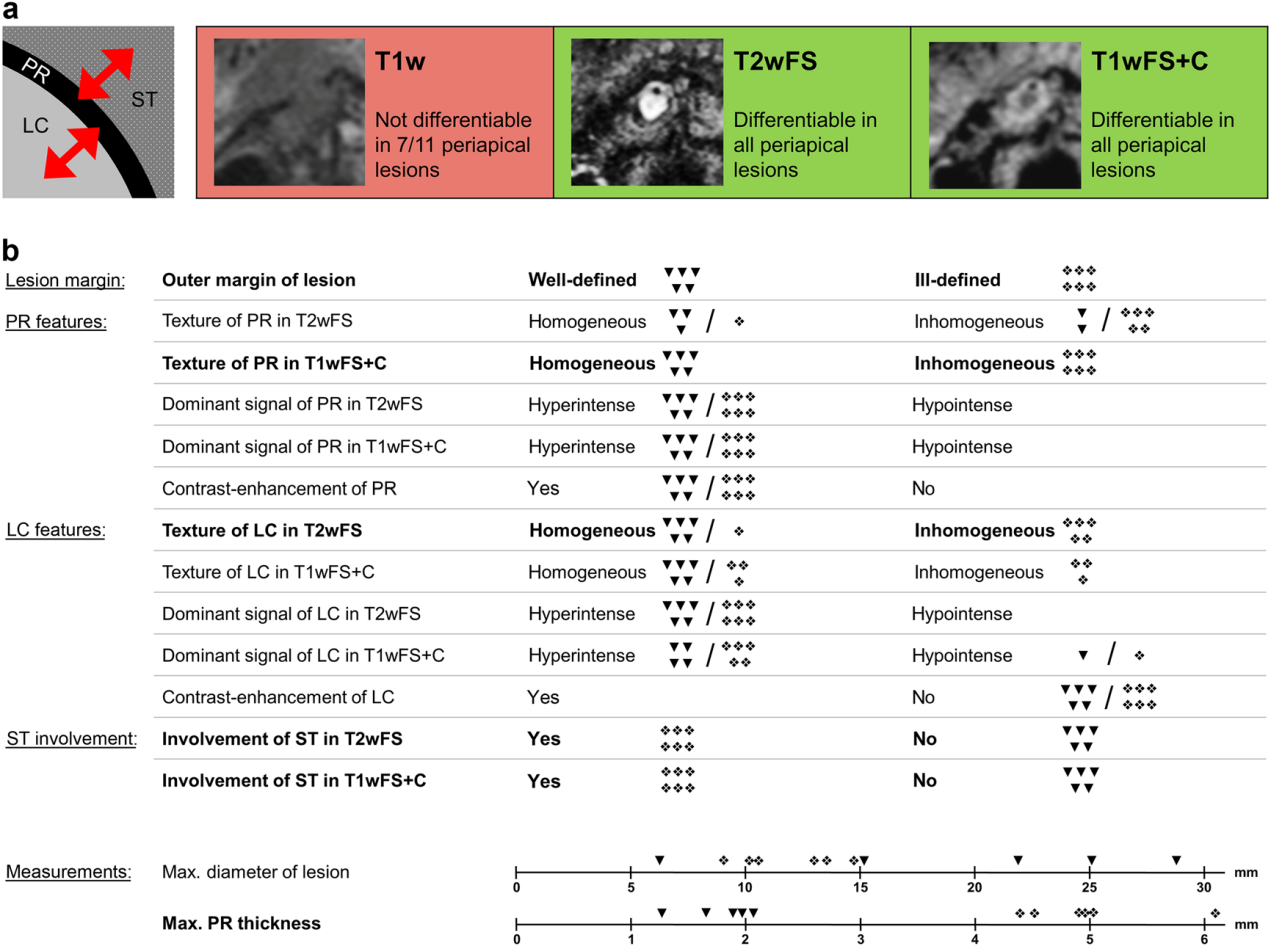
Study workflow and results for structured MRI analysis. **a** Differentiability of the peripherial rim in different MRI sequences.  **b** Morphometric MRI analysis on T2wFS and T1wFS+C images (distinguishing features are shown in bold text). T1w T1-weighted, T2wFS T2-weighted with fat saturation, T1wFS+C T1-weighted with fat saturation+contrast agent, PR peripheral rim, LC lesion center, ST surrounding tissue, ▼ cyst, ❖ granuloma

On non-contrast-enhanced T1-weighted (T1w) and non-contrast-enhanced fat-saturated T1-weighted (T1wFS) images, the lesions’ peripheral rim (PR) could not be distinguished from the surrounding tissue (ST) in 7/11 cases and the PR was not differentiable from the lesion center (LC) in 6/11 cases. However, fat-saturated T2-weighted (T2wFS) and contrast-enhanced fat-saturated T1-weighted (T1wFS+C) images allowed for a clear differentiation of the PR from the LC, as well as the ST in all lesions (Fig. 1a). T2wFS and T1wFS+C images were therefore used for structured analysis based on a total of 15 criteria (Fig. 1b).

For the reliability of maximum PR thickness measurements, intraclass correlation coefficient (ICC) values (95% confidence interval) were: intraobserver ICC observer 1 = 0.988 (0.945–0.997), intraobserver ICC observer 2 = 0.984 (0.945–0.996), interobserver ICC = 0.970 (0.836–0.993). For maximum lesion diameter, ICC values (95% confidence interval) were: intraobserver ICC observer 1 = 0.996 (0.987–0.999), intraobserver ICC observer 2 = 0.998 (0.992–0.999), interobserver ICC = 0.997 (0.988–0.999). The qualitative MRI parameters revealed an intraobserver reliability of Cohen’s kappa (κ) = 0.990 for observer 1 and κ = 0.995 for observer 2. Interobserver agreement of qualitative MRI parameters was κ = 0.988.

**Fig. 2:**
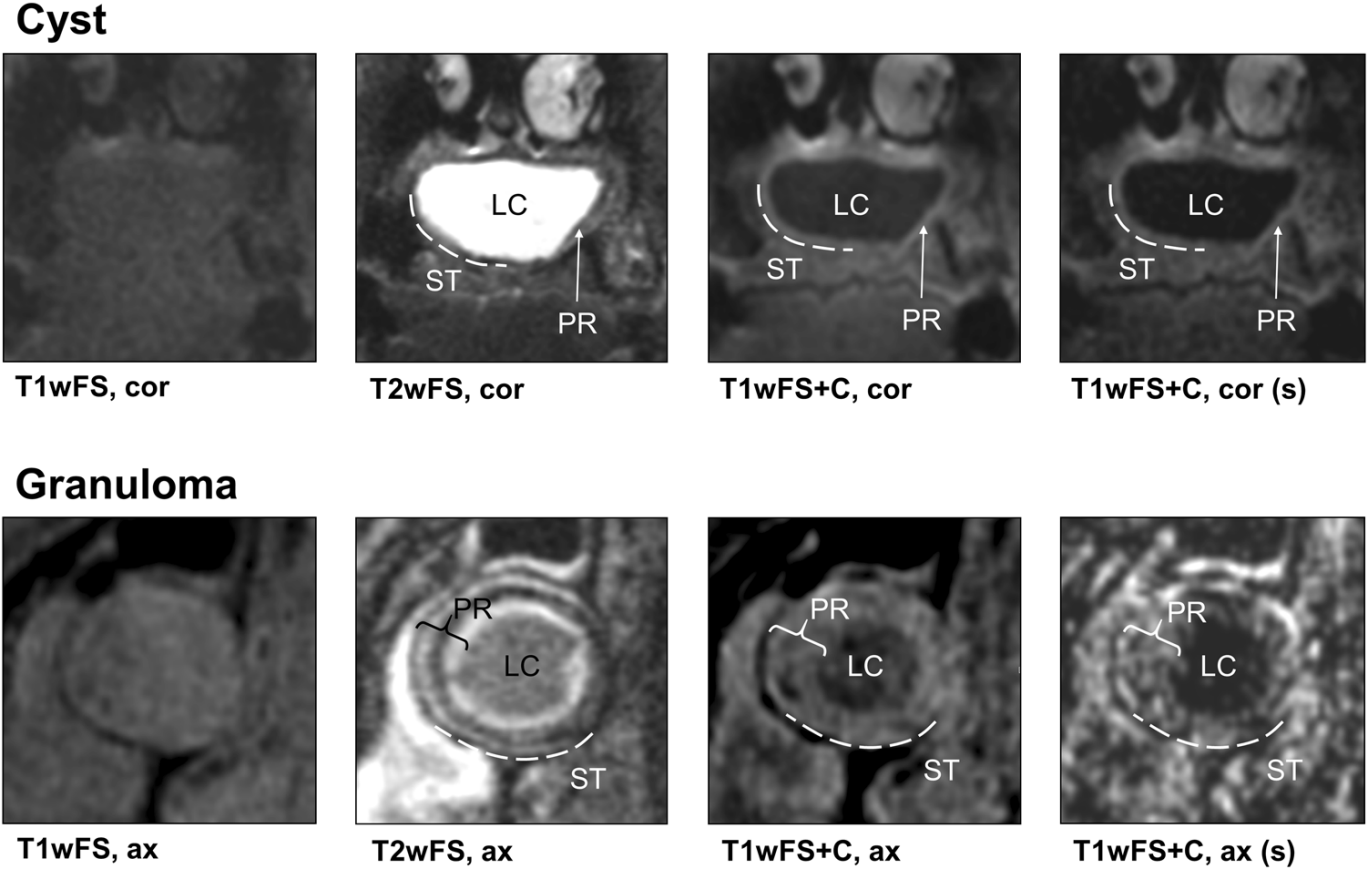
Typical MRI features of a periapical cyst and a granuloma. Unlike T2wFS and T1wFS+C, non-contrast-enhanced T1w/T1wFS images did not enable a reliable delineation of the peripheral rim. Six features allowed for differentiating between cysts and granulomas: (1) outer margin of the lesion (cysts: well-defined, granulomas: ill-defined). (2) Texture of the PR in T1wFS+C (cysts: homogeneous, granulomas: inhomogeneous). (3) Texture of the LC in T2wFS (cysts: homogeneous, granulomas: inhomogeneous). (4) Involvement of the ST in T2wFS (cysts: no, granulomas: yes). (5) Involvement of the ST in T1wFS+C (cysts: no, granulomas: yes) and (6) Maximum thickness of the PR (cysts: thin, granulomas: thick). T1wFS T1-weighted with fat saturation, T2wFS T2-weighted with fat saturation, T1wFS+C T1-weighted with fat saturation+contrast agent, (s) subtraction, cor coronal, ax axial, PR peripheral rim, LC lesion center, ST surrounding tissue

**Fig. 3:**
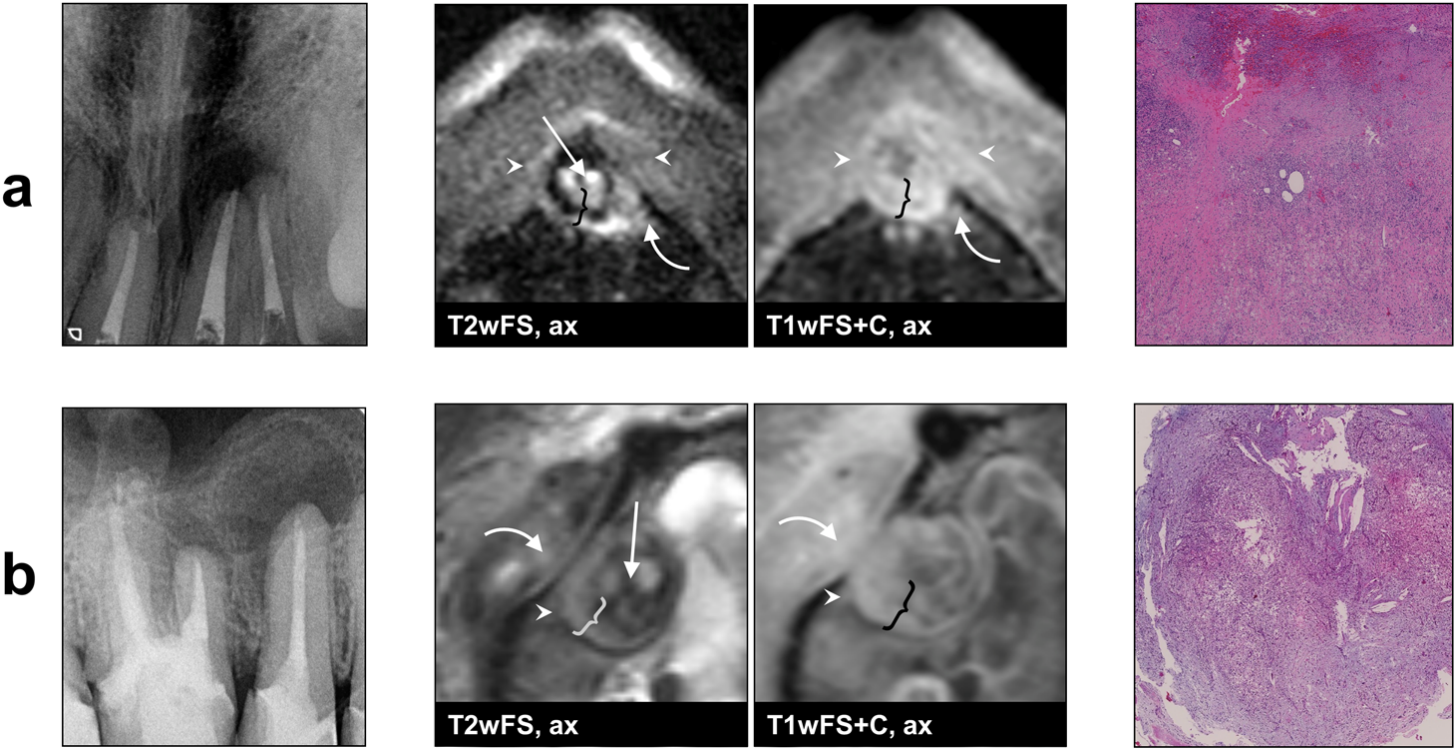
Example images of two periapical granulomas (radiographs, dental MRI and histopathology). Both lesions **a**, **b** were histologically confirmed as granulomas. On MRI, both granulomas show an ill-defined outer margin (arrow heads), an involvement the surrounding soft tissue in T2wFS and T1wFS+C (curved arrows), a wide peripheral rim with an inhomogeneous texture in T2wFS and T1wFS+C (curly brackets), and an inhomogeneous texture of the lesion center in T2wFS (straight arrows). T2wFS T2-weighted with fat saturation, T1wFS+C T1-weighted with fat saturation+contrast agent ax axial

**Fig. 4:**
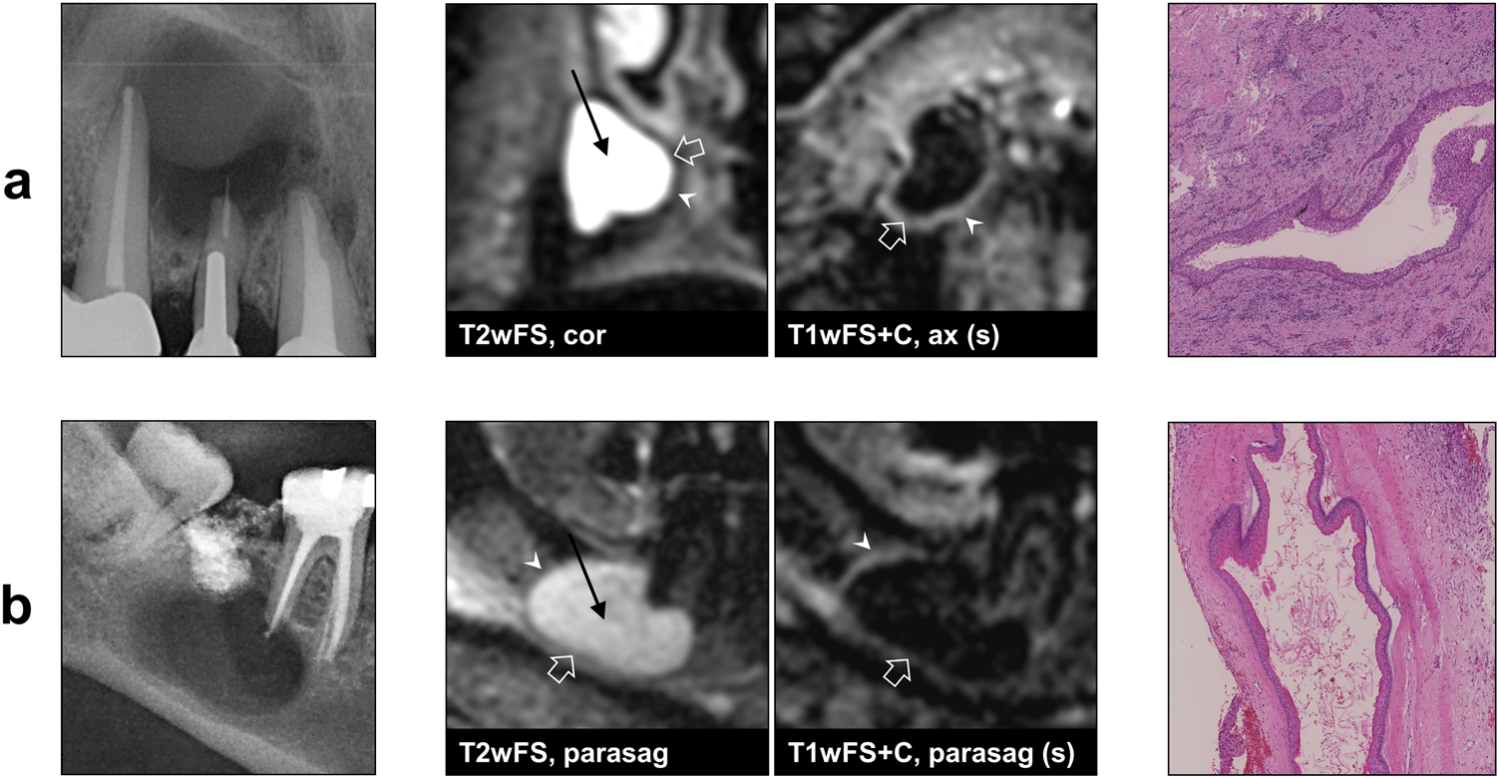
Example images of two periapical cysts (radiographs, dental MRI and histopathology). Histopathological results: **a**  radicular cyst; **b**  odontogenic keratocyst. On MRI, both cysts show a well-defined outer margin (arrow heads) without soft tissue involvement, a thin peripheral rim with a homogeneous contrast-enhancement in T1wFS+C (open arrows), and a homogeneous texture of the lesion center in T2wFS (straight arrows). Note the identical MRI features of the radicular cyst and the keratocyst. T2wFS T2- weighted with fat saturation, T1wFS+C T1-weighted with fat saturation+contrast agent, (s) subtraction, cor coronal, ax axial, parasag parasagittal

In accordance with histopathological results, a total of six MRI lesion characteristics allowed for a clear differentiation between cysts and granulomas in all cases: (1) outer margin of the lesion (cysts: well-defined, granulomas: ill-defined; *P* < 0.01), (2) texture of the PR in T1wFS+C (cysts: homogeneous, granulomas: inhomogeneous; *P* < 0.01), (3) texture of the LC in T2wFS (cysts: homogeneous, granulomas: inhomogeneous; *P* = 0.03), (4) involvement of the ST in T2wFS (cysts: no ST involvement, granulomas: ST involvement; *P* < 0.01), (5) involvement of the ST in T1wFS+C (cysts: no ST involvement, granulomas: ST involvement; *P* < 0.01) and (6) maximum thickness of the PR (cysts: thin, granulomas: thick; *P* = 0.01) with a mean value (± standard deviations (SD), range) of 1.6 mm (± 0.3 mm, 1.3–2.1 mm) for cysts and 4.6 mm (± 0.9 mm, 3.7–6.4 mm) for granulomas. Distribution of all diagnostic criteria is shown in Fig. 1, and *P*-values, sensitivity and specificity are given in Table 1. Figure 2 shows two cases outlining the typical MRI features of a periapical cyst and a periapical granuloma. In addition, two exemplary granulomas and two exemplary cysts including radiographs, MRI images and histological sections are illustrated in Figs. 3 and  4, respectively.

**Table 1:** Diagnostic criteria applied for the differentiation between periapical granulomas and cysts

Diagnostic criterion	*P*-value	Sens./Spec. granuloma/%	Sens./Spec. cyst/%
Outer margin of PR	**<0.01**	0/0 (well-defined) 100/100 (ill-defined)	100/100 (well-defined) 0/0 (ill-defined)
Texture of PR in T2wFS	0.27	17/40 (homogeneous) 83/60 (inhomogeneous)	60/83 (homogeneous) 40/17 (inhomogeneous)
Texture of PR in T1wFS+C	**<0.01**	0/0 (homogeneous) 100/100 (inhomogeneous)	100/100 (homogeneous) 0/0 (inhomogeneous)
Dominant signal of PR in T2wFS	n.a.*	100/0 (hyperintense) 0/0 (hypointense)	100/0 (hyperintense) 0/0 (hypointense)
Dominant signal of PR in T1wFS+C	n.a.*	100/0 (hyperintense) 0/0 (hypointense)	100/0 (hyperintense) 0/0 (hypointense)
Contrast-enhancement of PR	n.a.*	100/0 (yes) 0/0 (no)	100/0 (yes) 0/0 (no)
Texture of LC in T2wFS	**0.03**	17/0 (homogeneous) 83/100 (inhomogeneous)	100/83 (homogeneous) 0/17 (inhomogeneous)
Texture of LC in T1wFS+C	0.18	50/0 (homogeneous) 50/100 (inhomogeneous)	100/50 (homogeneous) 0/50 (inhomogeneous)
Dominant signal of LC in T2wFS	n.a.*	100/0 (hyperintense) 0/0 (hypointense)	100/0 (hyperintense) 0/0 (hypointense)
Dominant signal of LC in T1wFS+C	1.00	17/80 (hyperintense) 83/20 (hypointense)	20/83 (hyperintense) 80/17 (hypointense)
Contrast-enhancement of LC	n.a.*	0/0 (yes) 100/0 (no)	0/0 (yes) 100/0 (no)
Involvement of ST in T2wFS	**<0.01**	100/100 (yes) 0/0 (no)	0/0 (yes) 100/100 (no)
Involvement of ST in T1wFS+C	**<0.01**	100/100 (yes) 0/0 (no)	0/0 (yes) 100/100 (no)
Max. diameter of lesion	0.14	100/80 (τ ≤ 14.9 mm)	80/100 (τ ≥ 14.9 mm)
Max. PR thickness	**0.01**	100/100 (τ ≥ 3.2 mm)	100/100 (τ ≤ 3.2 mm)

Distinguishing features (FDR-adjusted *P*-values < 0.05) are shown in bold face

*T2wFS* T2-weighted with fat saturation, *T1wFS+C* T1-weighted with fat saturation+contrast agent, PR, peripheral rim, *LC* lesion center, *ST* surrounding tissue, *P-values cannot be computed because the variable is a constant, *Sens*.,  sensitivity, *Spec*. specificity, τ  threshold parameter in receiver operating characteristic (ROC) analysis

All other criteria investigated did not allow for differentiation of cysts and granulomas (Fig. 1b). For these criteria, we either observed mixed distribution or congruence of attributes for cysts and granulomas (*P* > 0.05 and n.a., respectively; Table 1). Cysts showed a larger average diameter (± SD, range) of 19.4 mm (± 8.7 mm, 6.1–28.4 mm) compared with 12.2 mm (± 2.2 mm, 9.4–15.2 mm) for granulomas, but the difference was not statistically significant (*P* = 0.14).

## Discussion

This pilot study was performed to evaluate whether dental MRI is able to differentiate between periapical cysts and granulomas. The major finding was that we identified six lesion characteristics clearly distinguishing between the two lesion types in accordance with histopathology. To the best of our knowledge, this is the first study reporting the statistical significance of MRI criteria for differentiating between periapical cysts and granulomas. Therefore, our results indicate that MRI could prove to be a reliable non-invasive diagnostic tool in apical periodontitis.

Several diagnostic attempts have been performed to differentiate periapical cysts from granulomas without surgical intervention, but none have proved to be reliable. It has been demonstrated that cysts cannot be differentiated from granulomas by evaluating the size and structure on radiographs.^15,16,36,37^ Early studies using computed tomography (CT)^38^ and CBCT^39^ yielded promising results, and further investigations revealed higher sensitivity of CBCT for detecting periapical lesions in comparison with radiographs.^40,41^ However, studies directly comparing CBCT with histopathological results could only show a moderate accuracy of CBCT in distinguishing cysts from granulomas.^20,21,42^ It should also be borne in mind that the added value of CBCT in comparison with conventional radiographs comes at the cost of a substantially increased radiation dose.^43^

One of the major advantages of MRI over CT and CBCT is its high soft tissue contrast and the possibility to vary the contrast by changing the MRI sequence design. More specifically, MRI not only provides an excellent soft tissue contrast, it also allows for the evaluation of specific tissue components in different sequence types before and after gadolinium administration. Given these strengths, radiation-free MRI has shown diagnostic superiority over CT techniques in various soft tissue-associated pathologies in the head and neck region.^44–46^ To date, however, only Geibel and colleagues have systematically analyzed apical bone lesions with MRI. In early 2015, they published a feasibility study evaluating the applicability of MRI for assessing apical periodontitis in 19 patients and compared MRI with CBCT images. By using conventional, non-contrast-enhanced T1w and T2w sequences at 3 Tesla, they demonstrated the feasibility of MRI for identifying periapical lesions and found MRI to be equal in sensitivity to CBCT. They also hypothesized that MRI could enable pre-interventional lesion characterization. In 2017, Geibel et al. investigated the potential of MRI for characterization of apical bone lesions in a case series. More precisely, they analyzed non-contrast-enhanced T1w and T2w images of 15 periapical lesions and compared the MRI findings with the corresponding CBCT images. Importantly, all lesions were analyzed histologically in this case series study. The investigators identified three criteria for possible lesion characterization: (I) texture of the lesion (homogeneous vs. heterogeneous), (II) signal intensity compared with the ST and (III) differences in signal intensity between T1w and T2w. Based on these criteria, they stated that MRI may lead to a better characterization of apical lesions. However, it is important to note that their data did not allow conclusions to be drawn regarding the differentiation between cysts and granulomas, because only 2 out of 15 lesions were cysts. In contrast, our study showed a ratio of five cysts vs. six granulomas, allowing for statistical analysis to investigate the distribution of the predefined MRI criteria. Another substantial difference between the data of Geibel et al. and our study is that we included fat-saturated, T2w sequences (T2wFS) and contrast-enhanced, fat-saturated, T1w sequences (T1wFS+C) in our protocol. Indeed, analysis of our study data confirmed the particular importance of contrast administration, as non-contrast-enhanced T1w images turned out not to be sufficient for the differentiation of the lesion against the STs in 7 out of 11 cases. Thus, only T2wFS and T1wFS+C images were suitable for our analysis method. Altogether, the favorable lesion ratio combined with the availability of T2wFS and T1wFS+C images allowed us to define 15 diagnostic MRI criteria, which could then be evaluated by two independent observers.

In the present study, 6 out 15 predefined MRI criteria revealed a statistically significant differentiation between cysts and granulomas, and these distinguishing features involved the LC, the PR and the ST of lesions:

- LC: in T2wFS, the texture of the LC was found to be homogeneous in cysts and inhomogeneous in granulomas. For cysts, our findings correspond very well with the liquid filled cavities, which are not vascularized.^47^ The heterogeneity in the LC of granulomas might be explained by the granulation tissue being infiltrated by (chronic) inflammatory cells.^48^
- PR: measurements of the maximum PR thickness allowed a clear differentiation between the two lesion types, with “thin-walled” cysts (mean: 1.6 mm) and “thick-walled” granulomas (mean: 4.6 mm). Another distinguishing feature was the PR texture in T1wFS+C, which was homogeneous in cysts and inhomogeneous in granulomas. The inhomogeneity of granulomas’ PR might be the correlate for infiltrates of lymphocytes, plasma cells and macrophages, which can be found on histological sections.^48^
- Lesion margin and ST: the outer margins of the lesions were well-defined in cysts and ill-defined in granulomas on MRI. In concordance with this, T2wFS as well as T1wFS+C images showed an involvement of the ST in granulomas, but not in cysts. Gaudino et al. postulated in 2011 that dental MRI could be useful in detecting inflammatory pathologies, which cannot be visualized by conventional radiographs, CT or CBCT at an early stage.^23^ In accordance with this, our findings indicate that the inflammation of a granuloma exceeds the outer margins defined by X-ray methods, leading to indistinct margins, as well as edema and contrast-enhancement of STs on MRI.

The remaining nine MRI criteria analyzed in this study did not allow for differentiation between cysts and granulomas. With regard to maximum lesion diameter, our findings are in line with a previous study performed on radiographs, which also reported that cysts are larger than granulomas on average, but that a differentiation based on this criterion is not possible due to the wide variation in both types of lesions.^36^ Interestingly, all granulomas analyzed in our study showed no contrast-enhancement in the LC. Thus, neovascularization^1^ seems to be significantly reduced in the central area of granulomas.

From the present study, it can be concluded that the MRI technique applied is essential for the analysis of periapical lesions, as these lesions have to be imaged sufficiently with regard to resolution, contrast, signal-to-noise ratio and susceptibility to artifacts. Therefore, various methodological aspects should be considered for future studies: (I) Coil system: an application-optimized coil system is of major importance. In the present study, we used a head and neck coil, providing a homogeneous signal distribution within the imaging volume.^24^ (II) Administration of contrast agent: our results confirmed that the diagnostic value of non-contrast-enhanced T1w images is very limited, as the lesion margins cannot be clearly delineated in many cases. In this context, it should also be noted that Geibel et al. stressed that non-contrast-enhanced T1w and T2w images could help to identify fluids, thus enabling the differentiation between cyst core and cyst wall.^34^ This was based on the assumption that fluids are hypointense in T1w and hyperintense in T2w on MRI. This may be the case for most cysts, but it must be borne in mind that the protein content in the lumen may be elevated, leading to a decrease in T1 relaxation time and—in turn—to an increase in T1w signal intensity. Results from our present study actually reflect this phenomenon, as we found a T1 hyperintense signal of the cyst lumen in two out of five cases. Altogether, results of this study indicate that contrast-enhanced T1w images play a pivotal role in the differentiation of periapical lesions. (III) Fat saturation: it should be pointed out that the T1w and T2w sequences applied in the present study included fat saturation, which facilitated the differentiation between the fatty bone marrow and the periapical lesions. (IV) Artifacts: another important aspect in dental MRI is the reduction of artifacts. In the present study, all sequences were optimized for short acquisition times to minimize the risk of motion artifacts. The best possible reduction of metal-induced artifacts is equally decisive, as ferromagnetic dental materials (especially when containing non-precious alloys) can cause severe artifacts.^49–51^ To prevent this, we used spin echo/turbo spin echo sequences, which have low susceptibility to metal-induced artifacts.^51,52^

The results of the present study provide a substantial diagnostic gain, because dental MRI allowed for a clear differentiation between periapical granulomas and periapical cysts. This may have a high clinical impact, as granulomas account for about 85% of all periapical lesions and are likely to heal with non-surgical root canal treatment.^4,6^ However, our data did not enable us to distinguish between different types of cysts and it remains unclear whether modern MRI techniques have the capacity to differentiate pocket cysts from true cysts. This is a limitation, as true cysts—in contrast to pocket cysts—are less likely to be healed by root canal therapy. Furthermore, one of the cysts in the present study was histologically classified as an odontogenic keratocyst. This non-inflammatory, neoplastic cyst formation had the same appearance as the other cysts, which were all radicular cysts. This suggests that the rarely occurring keratocysts^48,53^ can be misdiagnosed as radicular cysts on MRI, especially when they are small. Thus, according to our results and the current state of diagnostic possibilities, surgical therapy seems reasonable when a cyst is diagnosed on MRI. Potentially, three-dimensional (3D) sequences can be established for future MRI studies on periapical lesions. As these sequences yield isotropic datasets, they might be helpful for further differentiation between different cyst types. Furthermore, particularly in view of the minimization of partial volume effects, 3D sequences could provide substantial added assistance in diagnosing smaller lesions (< 5 mm) and might form the basis for computer-assisted quantitative analyses of surfaces and textures.

Although we identified six MRI criteria allowing for a clear differentiation between cysts and granulomas in this pilot project, results must be interpreted with caution due to the small number of evaluated periapical lesions. This particularly applies for measures of diagnostic accuracy, which should be assessed in future studies with larger patient numbers. If these criteria reveal to be highly sensitive and specific in larger and representative cohorts, dental MRI could be used as a non-invasive diagnostic tool to distinguish between cysts and granulomas. This could help to avoid unnecessary periapical surgery in many patients.

### Conclusions

In this pilot study, dental MRI allowed for the identification of six characteristics, which each have the capacity to clearly differentiate between periapical cysts and granulomas. Our findings may have a substantial clinical impact because they could contribute to improvements in diagnosis and avoidance of unnecessary surgery in patients with apical periodontitis. In the future, studies with larger patient numbers should be conducted to evaluate the robustness of our findings and to investigate whether periapical cysts can be further differentiated into pocket cysts, true cysts and non-inflammatory cystic lesions.

## Materials and methods

### Ethics

This prospective study was approved by the local research ethics committee of the University of Heidelberg (approval number: S-452/2010). Written informed consent was obtained from all participants.

### Patients

Eleven patients (three females and eight males) were prospectively enrolled in this study. Mean age was 39.5 years (range: 21–60 years). Inclusion criteria were a minimum age of 18 years and the diagnosis of a periapical radiolucency on intraoral dental radiography. Only patients with periapical lesions > 5 mm (based on radiographs) were considered for study enrollment. All included teeth were root canal treated before apical surgery. All patients with contraindications for apicoectomy or MRI contrast agents were excluded from the study.

### MRI examinations

All MRI examinations were performed prior to root-end resection on a 3T MRI system (MAGNETOM TIM Trio, Siemens Healthcare GmbH, Erlangen, Germany) with a 12-channel head coil and an additional matrix neck coil (Siemens Healthcare GmbH, Erlangen, Germany). The neck coil has a four-element design with two clusters of two elements each. Head and neck coils operate as receive-only coils. A dedicated dental MRI protocol was applied including a T2wFS, a T1w and a T1wFS sequence. T1w sequences give low signal intensities of tissues with high water content and facilitate high contrast images in combination with paramagnetic contrast agents. Contrast-enhancement reflects tissue vascularization and/or extravasation. T2w sequences provide an excellent soft tissue contrast determined by the water content. Fat saturation was applied, as fatty tissues appear bright in both T1w and T2w and can be confused with the bright signal of the contrast agent or high water concentration, respectively.

For each patient, T2wFS, T1w and T1wFS images were acquired without contrast-enhancement. Next, 0.1 mmoL^.^ kg^–1^ gadoterate (Gd) meglumine contrast agent (Dotarem®, Guerbet, France) was administered intravenously, before the T1wFS sequence was repeated. Identical pre- and post-contrast T1wFS images were digitally subtracted for subsequent data analysis. Contrast-enhanced T1wFS images are abbreviated as T1wFS+C in the following. Sequence parameters were: (I) T2wFS: turbo spin echo sequence, echo time: 87 ms, repetition time: 4580 ms, bandwidth: 90 Hz/pixel, number of averages: 2, echo train length: 9, flip angle: 150°, FoV: 150 × 150 mm^2^, acquisition matrix: 240 × 320, slice thickness: 2 mm, number of slices: 40, time of acquisition: 4:26 min. (II) T1w: spin echo sequence, echo time: 6.4 ms, repetition time: 680 ms, bandwidth: 248 Hz/pixel, number of averages: 1, echo train length: 1, flip angle: 90°, FoV: 168 × 210 mm^2^, acquisition matrix: 192 × 320, slice thickness: 2 mm, number of slices: 40, time of acquisition: 4:20 min. (III) T1wFS/T1wFS+C: spin echo sequence, echo time: 8.1 ms, repetition time: 700 ms, bandwidth: 159 Hz/pixel, number of averages: 2, echo train length: 1, excitation flip angle: 58°, FoV: 168 × 210 mm^2^, acquisition matrix: 218 × 320, slice thickness: 1.8 mm, number of slices: 19, time of acquisition: 3:18 min.

### Analysis of MRI datasets

All periapical lesions were analyzed with DICOM Imaging Software (Osirix v.7.0.3, Geneva, Switzerland). As a first step, we evaluated the differentiability of the lesions’ PR against the ST and the LC in T1w, T1wFS, T2wFS and T1wFS+C. As T1w and T1wFS images did not allow for a clear delineation in all cases (Fig. 1a), only T2wFS and T1wFS+C were included for further analysis. Next, a comprehensive set of diagnostic criteria including lesion size, lesion margin, PR features, LC features and involvement of ST was defined. In total, 15 diagnostic criteria (Fig. 1b) were analyzed by two independent observers (A.J. and T.H., both radiologists with 4 years’ experience in dental imaging), who performed image analysis twice on each periapical lesion with an interval of ≥ 8 weeks. The observers were blinded to their previous results, to each other, and to the final diagnosis (histopathology).

### Apical surgery and histopathological analysis

For all lesions included in the study, root-end resection was performed following routine clinical protocols. All teeth were root canal filled prior to apicoectomy. The surgical specimens obtained were sent to the Institute of Pathology at Heidelberg University Hospital. Tissue was fixed in paraffin, sliced and stained with hematoxylin–eosin before sections were analyzed by a specialized senior oral pathologist (C.F.) to confirm the diagnosis.

### Statistical analysis

Statistical analysis was performed using software (SPSS Version 24, IBM Corporation, Armonk, NY, United States). Intra- and interobserver agreement were analyzed by ICC for continuous variables and by κ for categorical variables. MRI characteristics of patients with granulomas and cysts were compared using the Mann–Whitney *U*-test for continuous variables and the Fisher's exact test for categorical variables. Resulting *p*-values were adjusted for multiple testing errors using the Benjamini and Hochberg false discovery rate (FDR) procedure. For determination of diagnostic accuracy, sensitivity and specificity were calculated, and receiver operating characteristic (ROC) analyses were performed for continuous variables.
